# Comparisons of financial hardship in cancer care by family structure and among those with and without minor children using nationally representative data

**DOI:** 10.1002/cam4.7088

**Published:** 2024-03-23

**Authors:** Patricia I. Jewett, Himal Purani, Rachel I. Vogel, Helen M. Parsons, Maria Borrero, Anne Blaes

**Affiliations:** ^1^ Department of Medicine University of Minnesota Minneapolis Minnesota USA; ^2^ Department of Obstetrics, Gynecology and Women's Health University of Minnesota Minneapolis Minnesota USA; ^3^ Department of Pediatrics University of Minnesota Minneapolis Minnesota USA; ^4^ Department of Neurology University of California Davis Davis California USA; ^5^ Division of Health Policy and Management University of Minnesota Minneapolis Minnesota USA

**Keywords:** cancer survivors, family structure, financial hardship, financial toxicity, parental status

## Abstract

**Introduction:**

While demographic risk factors of cancer‐related financial hardships have been studied, having minor children or being single have rarely been assessed in the context of healthcare‐related financial hardships.

**Methods:**

Using data from the 2015 to 2018 National Health Interview Survey, we assessed financial hardship (material and psychological hardship; behavioral coping due to costs: delaying/foregoing care, reducing prescription costs, or skipping specialists or follow‐up care) among adults aged 18–59 years with cancer (*N* = 2844) by minor child parenting status and family structure. In a secondary analysis, we compared this group with individuals without cancer. Using logistic regression models, we compared those with and without children aged <18 years, further distinguishing between those who were single versus one of two or more adults in the family.

**Results:**

Compared to individuals from families with two or more adults/without children, single adults with children more often reported cancer‐related financial hardships, for example material hardship (45.9% vs. 38.8%), and reducing prescription costs, (50.7% vs. 34.4%, adjusted OR 1.57, 95% CI 1.07–2.28). Single adults without minor children and those from families with two or more adults/with minor children also reported greater financial hardships on some dimensions. Associations were similar among those without cancer, but the overall magnitude of financial hardships was lower compared to those with cancer.

**Conclusions:**

Our findings suggest that having minor children, and being a single adult are risk factors for cancer‐related financial hardship. Financial vulnerability associated with family structure should be taken into consideration in healthcare, and especially cancer care.

## Graphical abstract

We used 2015–2018 National Health Interview Survey data to assess the associations of having minor children or being single with healthcare‐related financial hardships among adults aged 18–59 years with and without a history of cancer. Compared to individuals from families with two or more adults/without children, adults with different family structures, and especially single adults with children, more often reported cancer‐related financial hardships (measured as [material and psychological hardship; behavioral coping due to costs: delaying/foregoing care, reducing prescription costs, skipping specialists or follow‐up care]). While these associations of family structure with financial hardships were similar in those with and without cancer, the overall magnitude of financial hardship was highest among those with cancer.

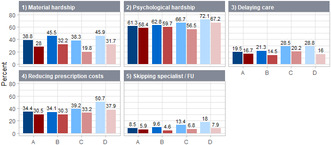

## 1 INTRODUCTION

The rising costs of healthcare are evident in all sectors of the healthcare industry, and especially within oncology. Due to the advent of novel cancer treatments, such as targeted chemotherapies and immunotherapies, which can cost more than $100,000 per year, individuals are living longer with cancer, experiencing cancer as a lifelong chronic illness. These survival improvements have come at high financial cost to cancer patients and their families.^1^, ^2^, ^3^

According to prior estimates, 15%–79% of cancer survivors experience healthcare‐related financial hardships, especially among low‐income populations.^4^, ^5^ Treatments that are covered by private or government‐issued insurance can still cost thousands of dollars annually in out‐of‐pocket costs related to deductibles, premiums, co‐pays, and out‐of‐network costs.^6^, ^7^ Contributors to financial burdens include direct medical costs (e.g., co‐pays, out‐of‐pocket costs for medications, treatments, and medical devices), indirect non‐medical costs (e.g., travel, parking, accommodation, childcare etc.) and opportunity costs (e.g., loss of income during treatment, loss of employment).^1^, ^4^ Adverse effects of these costs often continue long‐term due to disability, reduced employment,^7^, ^8^, ^9^ and stressed family dynamics (e.g., changed and increased caregiving roles).

Known risk factors of cancer‐related financial toxicity include being female, a person of color, younger age, having no health insurance, advanced cancer stage, and having a more recent diagnosis.^3^, ^4^, ^10^, ^11^ Risk factors may also include family structure such as being single and/or having minor children, but these factors have received less attention within research on healthcare‐related financial hardship. Models of wealth posit that wealth typically accumulates over the life course during working years and then gradually declines during retirement, but empirical research has shown that wealth distribution also strongly depends on family structure and size, with individuals with minor children or who are single typically being less wealthy.^12^ Adults with young children likely have higher‐than‐average family‐related ongoing household expenses, and since parents/guardians of young children are usually young adults themselves, they have had less time to accumulate wealth, and often have large financial obligations such as mortgages, childcare costs, college costs for older siblings etc.^13^ Further, whether families are financially supported by one or several incomes likely matters with regard to financial vulnerability.

Therefore, the aim of our research was to describe cancer care‐related financial hardships among adults with cancer, comparing by family structure, i.e., comparing those with families that do or do not include minor children (aged 17 years or younger), and those with only one adult versus with two or more adults in the family. In a secondary analysis, we also assessed the magnitude of financial hardships and the associations of family structure with financial hardships in adults without cancer. We hypothesized that financial hardships would be more frequently reported by individuals with families with minor children compared to individuals without minor children, and more often if adults were single compared to one of two or more adults in the family. Furthermore, we hypothesized that we would detect these patterns of financial hardships by family structure both among adults with and without cancer, but that financial hardship would be overall more severe among those with cancer.

## 2 MATERIALS AND METHODS

### 2.1 Data source and study population

Data were obtained from 2015 to 2018 National Health Interview Surveys (NHIS).^14^ These data are publicly available, and the study was thus deemed exempt by the University of Minnesota Institutional Review Board. The NHIS is a national survey conducted by the U.S. Census Bureau on behalf of the National Center for Health Statistics (NCHS). The primary objective of the NHIS is to monitor the health of the civilian, noninstitutionalized U.S. population by collecting and analyzing data on a broad range of topics, facilitating analyses by demographic and socioeconomic characteristics. Data are collected annually through personal household interviews. We restricted the NHIS data to non‐elderly adults aged 59 or younger to focus on a population most likely to have children aged 17 or younger. We distinguished between individuals with versus without a personal history of cancer using self‐reported information on ever having been diagnosed with cancer. Individuals with non‐melanoma skin cancer only and no other cancers were assigned to the non‐cancer group.

### 2.2 Measures

Financial hardship was measured on several sub‐dimensions: material financial hardship (problems paying bills, paying bills over time), psychological financial hardship (being worried about ability to pay for medical bills and health care), and behavioral coping to avoid medical costs, including (1) delaying or foregoing care, (2) reducing prescription costs (skipping doses, taking less medicine, delaying prescriptions, asking for lower cost medication or cheaper drugs from other countries, alternative therapy to save money), and (3) skipping follow‐up or specialist care. The exact wording of the NHIS survey questions used to measure financial hardship, and the logic for how we operationalized these questions to generate the binary (yes/no) outcomes for our analysis are shown in Table .

Family structure was defined based on (1) self‐reported family size, and (2) self‐reported number of family members under the age of 18 years. Based on these data, we defined whether participants lived with children aged 17 years or younger (“minor children”) versus not, and how many adults were part of the family (family size minus number of children under the age of 18 years) to create a categorical exposure variable with four levels: (1) individuals from families with two or more adults/without minor children; (2) individuals from families with two or more adults/with minor children; (3) individuals who reported being the only adult in the family (referred to as “single adults” in this manuscript)/without minor children; and (4) single adults with minor children. In our statistical comparisons, we utilized individuals from families with two or more adults/without minor children as the reference group based on our hypothesis that these individuals faced the lowest risk of financial hardship given their family structure.

We added an additional intersectional sub‐analysis by divorced/separated marital status (yes/no) because partner splitting, while frequent, may aggravate financial stressors. Divorced/separated marital status was not included as covariate in the main models because it was too closely associated with the main exposure (family structure).

### 2.3 Statistical analysis

Descriptive statistics were used to describe demographic characteristics of the study population, by cancer status and family structure (four‐level variable). We report survey‐weighted frequencies and proportions, and used unadjusted and adjusted logistic regression models to calculate odds ratios of greater financial hardship by family structure. We included the following confounders in the logistic regression models (identified a priori): age (years), sex (male vs. female), race/ethnicity (Hispanic vs. non‐Hispanic [NH] Black vs. NH White vs. NH other), and education (no high school degree vs. no college degree versus at least a college degree). We chose to not include income or health insurance coverage as covariates, since we expected these factors to be mediators along the family structure—financial hardship pathway. All analyses were stratified by participants' cancer status and we accounted for NHIS survey weights. We used SAS 9.4 for our analyses.^15^

## 3 RESULTS

### 3.1 Characteristics of the study population

At total of 2844 cancer survivors were included in our analysis, of which 68.1% were female (Table 1). The mean age was 47.7 years (median 50 years, interquartile range 40.9–55.2). About a quarter (28.9%) reported being single adults without minor children, 8.7% reported being single adults with minor children, 38.3% reported there were two or more adults in the family/without minor children, and 24.1% reported there were two or more adults in the family/with minor children. Individuals with minor children were younger (35.8% aged 39 years or younger among those with two or more adults in the family, 37.8% among single adults), compared to those without minor children (11.0% aged 39 years or younger among those with two or more adults in the family, 16.8% among single adults). The share of non‐Hispanic White participants was highest among those with two or more adults in the family/without minor children (81.0%), and lowest among single adults with minor children (65.9%). Single adults with minor children were more likely to be NH Black (17.7%) or Hispanic (14.3%) than adults with other family structures. A large majority of single adults with minor children were female (85.7%). Household incomes were highest among those with two or more adults in the family/without minor children (56.6% with incomes greater or equal 4 times the poverty line [PL]; 8.4% below PL); and lowest among single adults with minor children (12.3% with incomes greater or equal 4 times the PL; 39.6% below PL). Education levels were highest among those with two or more adults in the family/with minor children (48.4% with at least a college degree), and lowest among single adults with minor children (22.1% with at least a college degree).

**TABLE 1:** Characteristics of individuals diagnosed with cancer by family status, restricted to participants aged <60 years, survey‐weighted frequencies, *N* = 2844, National Health Interview Surveys (NHIS) 2015–2018.

Characteristic	Everyone (*N* = 2844) % (95% CI)	Two or more adults in family/without minor children (*N* = 1082) % (95% CI)	Two or more adults in family/with minor children (*N* = 694) % (95% CI)	Single adult in family/without minor children (*N* = 822) % (95% CI)	Single adult in family/with minor children (*N* = 246) % (95% CI)
Age group					
<40	21.0 (19.2, 22.7)	11.0 (9.0, 13.0)	35.8 (32.2, 39.4)	16.8 (13.8, 19.8)	37.8 (30.7, 44.9)
40–49	24.2 (22.5, 25.9)	16.2 (13.7, 18.7)	38.2 (34.6, 41.7)	20.6 (17.9, 23.2)	33.1 (26.6, 39.6)
50–59	54.8 (52.8, 56.7)	72.8 (69.8, 75.8)	26.0 (22.6, 29.5)	62.6 (59.2, 66.0)	29.1 (23.1, 35.0)
Sex					
Female	68.1 (66.1, 70.1)	68.1 (64.9, 71.2)	70.9 (67.3, 74.4)	60.6 (57.1, 64)	85.7 (81.3, 90.1)
Male	31.9 (29.9, 33.9)	31.9 (28.8, 35.1)	29.1 (25.6, 32.7)	39.4 (36.0, 42.9)	14.3 (9.9, 18.7)
Race/ethnicity					
Hispanic	8.7 (7.5, 9.9)	7.3 (5.6, 9.1)	9.6 (7.3, 11.8)	8.2 (6.0, 10.4)	14.3 (9.8, 18.8)
Non‐Hispanic Black	9.0 (7.7, 10.3)	7.2 (5.3, 9.0)	5.3 (3.2, 7.3)	12.0 (9.5, 14.5)	17.7 (12.2, 23.3)
Non‐Hispanic other	3.9 (3.1, 4.8)	4.5 (3.0, 6.0)	5.2 (3.2, 7.2)	2.6 (1.5, 3.7)	2.1 (0.2, 3.9)
Non‐Hispanic White	78.3 (76.4, 80.3)	81.0 (78.2, 83.7)	80.0 (76.5, 83.4)	77.2 (74.0, 80.5)	65.9 (59.3, 72.6)
Income to poverty line (PL) ratio					
<PL	17.5 (15.9, 19.2)	8.4 (6.6, 10.3)	11.0 (8.7, 13.3)	28.1 (24.7, 31.5)	39.6 (32.9, 46.2)
1 to 2 × PL income	16.5 (15.0, 18.1)	11.0 (8.9, 13.0)	17.9 (14.9, 20.9)	18.7 (16.0, 21.5)	29.1 (23.3, 35.0)
2 to 4 × PL income	23.1 (21.3, 24.9)	23.9 (21.1, 26.8)	27.3 (23.6, 30.9)	19.7 (16.8, 22.6)	19.1 (13.7, 24.5)
≥4 × PL income	42.8 (40.5, 45.2)	56.6 (53.3, 60.0)	43.8 (39.4, 48.3)	33.5 (29.6, 37.3)	12.3 (8.1, 16.4)
Education					
No high‐school degree	9.0 (7.8, 10.2)	6.2 (4.6, 7.9)	6.7 (4.9, 8.4)	12.3 (10.1, 14.5)	16.6 (11.5, 21.6)
No college degree	50.1 (47.9, 52.3)	49.5 (46.1, 52.8)	45.0 (41.1, 48.8)	51.9 (48.1, 55.6)	61.4 (54.4, 68.4)
At least college degree	40.9 (38.5, 43.3)	44.3 (41.0, 47.6)	48.4 (44.3, 52.5)	35.8 (32.1, 39.5)	22.1 (16.1, 28.0)

### 3.2 Financial hardship by family structure among cancer survivors

**FIGURE 1:**
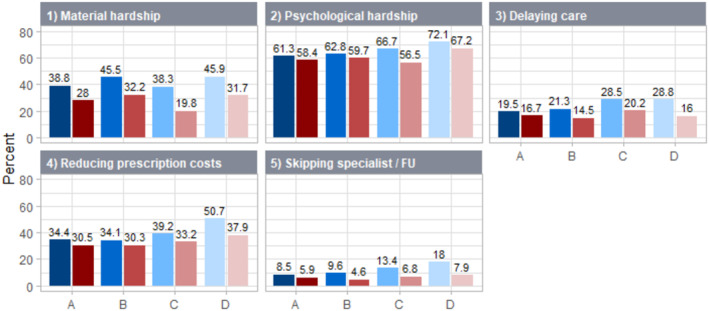
Financial hardships among those with (blue) and without (red) cancer: National Health Interview Surveys (NHIS) 2015–2018 restricted to participants aged <60 years. (A) Individuals with two or more adults in family/without minor children. (B) Individuals with two or more adults in family/with minor children. (C) Individuals who are the single adult in family/without minor children. (D) Individuals who are the single adult in family/with minor children.

With individuals from families with two or more adults/without minor children as the reference group, single adults with minor children more often reported financial hardship on almost all dimensions, for example, reducing prescription costs, adjusted OR 1.57, 95% CI 1.07–2.28, and delaying/foregoing care, unadjusted OR 1.63, 95% CI 1.16–2.29 (Table 2). Except for reducing prescription costs, these associations were no longer significant after adjusting for age, sex, race/ethnicity, and education. Single adults without minor children also more often reported financial hardship, for example delaying/foregoing care, adjusted OR 1.60, 95% CI 1.26–2.03, and psychological hardship, adjusted OR 1.26, 95% CI 1.02–1.56. Individuals from families with two or more adults/with minor children more often reported material hardship (adjusted OR 1.31, 95% CI 1.05–1.65). The frequency of material hardship was highest among those with minor children (single adults 45.9%; individuals from families with two or more adults 45.5%, Figure 1). Other financial hardships (psychological hardship, delaying/foregoing care, reducing prescription costs, skipping follow‐up or specialist care) were least frequent among individuals from families with two or more adults/without minor children, followed by individuals from families with two or more adults/with minor children, single adults without children, and were most common among single adults with minor children.

**TABLE 2:** Associations of family structure with financial hardship among those diagnosed with cancer; unadjusted and adjusted odds ratios (OR and aOR); National Health Interview Surveys (NHIS) 2015–2018 restricted to participants aged <60 years.

Outcome	OR (95% CI)	*p*	aOR^a^ (95% CI)	*p*
Material hardship				
Two or more adults in family/without minor children	1	(Ref.)	1	(Ref.)
Two or more adults in family/with minor children	**1.31 (1.05, 1.65)**	**0.02**	**1.30 (1.02, 1.65)**	**0.03**
Single adult in family/without minor children	0.98 (0.79, 1.21)	0.83	0.90 (0.72, 1.13)	0.36
Single adult in family/with minor children	1.34 (0.96, 1.86)	0.09	0.98 (0.69, 1.39)	0.91
Psychological hardship				
Two or more adults in family/without minor children	1	(Ref.)	1	(Ref.)
Two or more adults in family/with minor children	1.06 (0.85, 1.32)	0.59	1.13 (0.88, 1.45)	0.32
Single adult in family/without minor children	**1.26 (1.03, 1.56)**	**0.03**	**1.26 (1.02, 1.56)**	**0.03**
Single adult in family/with minor children	**1.63 (1.16, 2.29)**	**0.005**	1.37 (0.95, 1.96)	0.09
Delaying/foregoing care				
Two or more adults in family/without minor children	1	(Ref.)	1	(Ref.)
Two or more adults in family/with minor children	1.12 (0.88, 1.44)	0.36	1.10 (0.83, 1.44)	0.51
Single adult in family/without minor children	**1.65 (1.31, 2.08)**	**<0.0001**	**1.60 (1.26, 2.03)**	**0.0001**
Single adult in family/with minor children	**1.67 (1.15, 2.43)**	**0.007**	1.33 (0.89, 1.98)	0.16
Reducing prescription costs				
Two or more adults in family/without minor children	1	(Ref.)	1	(Ref.)
Two or more adults in family/with minor children	0.98 (0.77, 1.25)	0.88	0.91 (0.71, 1.17)	0.45
Single adult in family/without minor children	1.23 (0.98, 1.54)	0.08	1.21 (0.96, 1.52)	0.11
Single adult in family/with minor children	**1.95 (1.38, 2.77)**	**0.0002**	**1.57 (1.07, 2.28)**	**0.02**
Skipping specialist or follow‐up care				
Two or more adults in family/without minor children	1	(Ref.)	1	(Ref.)
Two or more adults in family/with minor children	1.13 (0.79, 1.63)	0.50	0.92 (0.61, 1.36)	0.66
Single adult in family/without minor children	**1.66 (1.15, 2.38)**	**0.006**	**1.56 (1.06, 2.29)**	**0.02**
Single adult in family/with minor children	**2.35 (1.38, 4.01)**	**0.002**	1.55 (0.87, 2.77)	0.14

^a^ Adjusted for age, sex, race/ethnicity, and education.

p <.05 was considered significant.

### 3.3 Comparisons of financial hardship by family structure and cancer status

While our main analysis was restricted to those with cancer, we tested for interaction between cancer status and family structure in additional models including all participants regardless of cancer status. These interaction terms were significant for the outcomes material financial hardship (*p* = 0.001), delaying/foregoing care (*p* = 0.01), and skipping follow‐up or specialist care (*p* = 0.04). The distribution of demographic characteristics was similar among those without cancer compared to those with cancer, with highest poverty and lower education levels, and higher proportions of persons of color among single adults/with children; however, those without cancer were overall younger than those with cancer (Table S2). General patterns of financial hardship by family structure were similar among individuals with or without cancer, but the overall magnitude of financial hardships was lower among those without cancer across all sub‐dimensions (Table 3 and Figure 1). Among individuals without cancer, single adults with minor children more often reported material and psychological financial hardship and reducing prescription costs than individuals from families with two or more adults/without minor children. However, effect sizes of these associations were smaller than among those with cancer, for example reducing prescription costs, adjusted OR 1.21, 95% CI 1.10–1.33 (Table 3). Further, there were some mixed findings among those without cancer: for example, individuals from families with two or more adults/with minor children were less often reported foregoing care, adjusted OR 0.84, 95% CI 0.80–0.90.

**TABLE 3:** Associations of family structure with financial hardship among those without cancer; unadjusted and adjusted odds ratios (OR and aOR); National Health Interview Surveys (NHIS) 2015–2018 restricted to participants aged <60 years.

Outcome	OR (95% CI)	*p*	aOR^a^ (95% CI)	*p*
Material hardship				
Two or more adults in family/without minor children	1	(Ref.)	1	(Ref.)
Two or more adults in family/with minor children	**1.22 (1.16, 1.28)**	**<0.0001**	**1.28 (1.22, 1.35)**	**<0.0001**
Single adult in family/without minor children	**0.63 (0.60, 0.67)**	**<0.0001**	**0.60 (0.56, 0.63)**	**<0.0001**
Single adult in family/with minor children	**1.19 (1.10, 1.29)**	**<0.0001**	0.95 (0.87, 1.03)	0.18
Psychological hardship				
Two or more adults in family/without minor children	1	(Ref.)	1	(Ref.)
Two or more adults in family/with minor children	**1.06 (1.01, 1.10)**	**0.01**	**1.05 (1.01, 1.10)**	**0.02**
Single adult in family/without minor children	**0.93 (0.88, 0.98)**	**0.004**	**0.94 (0.89, 0.99)**	**0.02**
Single adult in family/with minor children	**1.46 (1.36, 1.58)**	**<0.0001**	**1.20 (1.12, 1.30)**	**<0.0001**
Delaying/foregoing care				
Two or more adults in family/without minor children	1	(Ref.)	1	(Ref.)
Two or more adults in family/with minor children	**0.85 (0.80, 0.90)**	**<0.0001**	**0.84 (0.80, 0.90)**	**<0.0001**
Single adult in family/without minor children	**1.26 (1.19, 1.34)**	**<0.0001**	**1.20 (1.13, 1.28)**	**<0.0001**
Single adult in family/with minor children	0.96 (0.87, 1.05)	0.36	**0.76 (0.69, 0.84)**	**<0.0001**
Reducing prescription costs				
Two or more adults in family/without minor children	1	(Ref.)	1	(Ref.)
Two or more adults in family/with minor children	0.99 (0.93, 1.05)	0.68	1.01 (0.95, 1.08)	0.71
Single adult in family/without minor children	**1.13 (1.07, 1.20)**	**<0.0001**	**1.12 (1.06, 1.19)**	**0.0001**
Single adult in family/with minor children	**1.39 (1.27, 1.52)**	**<0.0001**	**1.21 (1.10, 1.33)**	**<0.0001**
Skipping specialist or follow‐up care				
Two or more adults in family/without minor children	1	(Ref.)	1	(Ref.)
Two or more adults in family/with minor children	**0.77 (0.69, 0.86)**	**<0.0001**	**0.78 (0.70, 0.87)**	**<0.0001**
Single adult in family/without minor children	**1.15 (1.04, 1.27)**	**0.006**	**1.14 (1.03, 1.26)**	**0.01**
Single adult in family/with minor children	**1.36 (1.16, 1.58)**	**0.0001**	0.99 (0.84, 1.16)	0.87

^a^ Adjusted for age, sex, race, and education.

p <.05 was considered significant.

### 3.4 Intersectional analysis to assess the role of partner splitting/divorce

In an intersectional comparison by age group, divorced/separated marital status, and by minor children, financial hardship was consistently higher among those who were divorced/separated compared to those not divorced/separated within each corresponding age/minor children category (Table S3). In this intersectional comparison, adults with cancer aged >59 years were included since no positivity assumptions regarding child‐rearing status needed to be made. While financial burdens were slightly lower in adults aged 40–59 years compared to those <40 years, they were dramatically lower in those aged 60 years and older.

## 4 DISCUSSION

The results largely aligned with our hypotheses: prevalence of financial hardship differed by family structure and was especially frequent among single adults with minor children. Compared with individuals from families with two or more adults/without minor children, those who were single without minor children and those from families with two or more adults/with minor children also reported more frequent care‐related financial hardships on some financial hardship dimensions captured in this study. The overall magnitude of financial hardships and effect sizes of the associations of family structure with care‐related financial hardships were higher among those with cancer than among those without cancer. The associations were attenuated after adjusting for age, sex, race/ethnicity, and education. An intersectional sub‐analysis highlighted the special case of divorce/separation as a potentially aggravating, while common factor with regard to financial hardship in single‐adult households.

Our findings add to and are consistent with previous studies of financial burdens of cancer, highlighting the high prevalence of material and psychological financial hardship in some cancer populations, and of coping strategies such as taking steps to reduce prescription costs or foregoing care in order to avoid costs that these activities incur.^2^, ^3^, ^16^, ^17^, ^18^ Our study emphasizes the high prevalence of financial hardships among cancer patients who have minor children, particularly among single parents. Parenting of minor children is rarely studied as a risk factor for healthcare‐related financial hardship; our findings are consistent with the few studies on that topic.^19^, ^20^ The observation that healthcare‐related financial hardships are more grievous in families with minor children, particularly among those with cancer, is perhaps unsurprising: cancer patients may have to reduce or quit work, at least temporarily, and in addition to income, they may lose insurance from their work.^21^, ^22^ Furthermore, cancer patients with minor children may have additional childcare needs adding to their costs.^20^ In our study population, 39.6% of single adult cancer patients with minor children reported household incomes below the PL, an indicator that these individuals and their families are at great risk of healthcare‐induced financial stress. The unique burdens of cancer patients who have minor children are seldom raised or addressed in research or clinical practice. Beyond those with minor children, single adults without minor children also more often reported financial hardships compared to individuals from families with two or more adults/without minor children, pointing to those without multiple income sources as another vulnerable group.

Some research groups have assessed the impact of cancer on parents with minor children. One focus of these studies has been the double psychological burden of parents diagnosed with cancer, who not only must face their own existential fears after their diagnosis—especially if the prognosis is grim—but also concerns and fears for their children.^23^, ^24^, ^25^ Many parents with cancer feel less able to meet their children's needs, and some parents have to contemplate the possibility that they might die before their children reach adulthood.^23^, ^26^, ^27^, ^28^ Our study adds to this literature by shedding light on financial stressors as yet another source of cancer burden with which families with minor children disproportionately struggle.

We ran a secondary analysis by family structure among those without cancer because we believed that patterns of financial hardships by family structure are a larger societal phenomenon not entirely unique to cancer.^29^, ^30^, ^31^ Healthcare‐related financial hardships have also been described in the context of other health conditions^32^ and as a potential burden in the general population.^33^ We confirmed that associations of family structure with financial hardship were similar among those with and without cancer, but the overall magnitude of financial hardship was greatest among those with cancer. As such, financial hardship from cancer care by family structure might be viewed through an intersectional lens: while following systemic patterns of how financial hardship is generally distributed in the U.S. depending on whether one is a single adult and whether one has minor children, the magnitude of financial hardship is unique when an expensive disease such as cancer is an added complication.

Some associations of family structure with financial hardships became statistically nonsignificant after adjusting for age, sex, race/ethnicity, and education among those with cancer suggesting that demographic factors partially explained the observed associations. However, many associations remained significant and similar, and in other cases, borderline significant. It is possible that the small sample size in some of the cancer subgroups, e.g., *N* = 246 among single adults with minor children and *N* = 694 among those with two or more adults in the family/with minor children, reduced our power to detect independent effects of family structure after adjusting for covariates. The respective sample sizes in the non‐cancer subgroups were *N* = 5504 and *N* = 24,618, and in these groups, the associations remained significant without much attenuation after adjustment for demographic factors. Another possible reason for greater effect attenuation by demographic factors among those with cancer is that the influence of factors such as gender and race/ethnicity may be greater when costs are higher, i.e., predisposing demographic risk factors may play out more strongly given the more drastic financial circumstances created by expensive cancer treatments.

We do not have an immediate explanation for the mixed findings with regard to some of the financial hardship sub‐dimensions among those without cancer. For example, contradicting our hypotheses, individuals without cancer from families with two or more adults/with minor children were less often reported foregoing care or skipping follow‐up or specialist care than adults from families with two or more adults/without minor children. Possible explanations why having children was associated with greater adherence to care appointments among families unaffected by cancer include: participants with minor children may have included well‐child visits in how they answered these questions, and some participants may simply have had no need for such services because they were healthy.

## 5 STRENGTHS AND LIMITATIONS

A strength of our study was the use of a nationally representative study population of the NHIS survey with a relatively large sample size that allowed for stratifying by cancer status and family structure. Our findings may also have implications beyond the current study. We focused on individuals who themselves have a history of cancer. As cancer‐related financial hardships likely affect the whole family, future studies should examine the role of these vulnerable family structures among caregivers of adult or minor children with cancer, which may provide insights into the unique circumstances and burdens of individuals who care for young dependents. Limitations include the cross‐sectional nature of the data which prohibit causal interpretations. Further, even after combining several NHIS survey waves, among those with cancer, some of the groups of interest had limited sample size (e.g., single adults with minor children, *N* = 246). Sample size and the way that race and ethnicity are captured within NHIS prohibited further stratification by race and ethnicity, which may be pertinent if family status and ethnoracial identity intersect with regard to cancer‐related financial hardships. We contrasted adults diagnosed with cancer with those never diagnosed with cancer, but the non‐cancer group likely included individuals with other conditions that come with high out‐of‐pocket medical costs which may have increased financial hardship estimates among those without cancer.^34^ Further, within the cancer group, we did not adjust for time since diagnosis. Financial burdens may be most acute closer to diagnosis while also persisting at some level with time. A global comparison of all cancer survivors with individuals who never had cancer was intentional in our study; future work should assess potential reductions in cancer‐related financial burden with time by family structure in greater detail. Social desirability and stigma related to poverty may have biased the way some participants self‐reported some of the NHIS outcomes utilized in our study.

## 6 CONCLUSION

Family structure is rarely conceptualized and assessed as a risk factor for healthcare outcomes, including for financial hardship following a cancer diagnosis. We observed that adults with minor children and single adults faced greater financial burdens than adults from families with two or more adults/without minor children, and the severity of those financial hardships was greatest among those with cancer. These potential financial vulnerabilities of patients and their families should be taken into consideration when providing healthcare, especially in cancer care. More research is needed on how family structure interacts with other risk factors, for example, race and ethnicity, in relation to healthcare‐related financial burdens. Finding ways to alleviate financial burdens of healthcare and particularly cancer care on young families and single adults are needed, for example by providing free childcare in cancer clinics,^20^ through policies for paid leave,^1^, ^35^, ^36^ expansion of programming to ensure access to insurance, and honest provider‐patient conversations about effects of cancer treatments on families' financial well‐being.

## AUTHOR CONTRIBUTIONS

**Patricia I. Jewett:** Conceptualization (equal); formal analysis (lead); investigation (lead); methodology (lead); visualization (lead); writing – original draft (equal); writing – review and editing (equal). **Himal Purani:** Conceptualization (equal); formal analysis (supporting); investigation (equal); methodology (supporting); writing – original draft (equal); writing – review and editing (supporting). **Rachel I. Vogel:** Conceptualization (supporting); formal analysis (supporting); investigation (supporting); methodology (supporting); writing – review and editing (equal). **Helen M. Parsons:** Conceptualization (supporting); investigation (supporting); methodology (supporting); writing – review and editing (supporting). **Maria Borrero:** Formal analysis (supporting); investigation (supporting); writing – review and editing (supporting). **Anne Blaes:** Conceptualization (equal); investigation (supporting); supervision (equal); writing – review and editing (supporting).

## CONFLICT OF INTEREST STATEMENT

None of the authors have any disclosures or conflicts of interests to declare.

## PRECIS

Parental status and family structure seldom are considerations in cancer care. However, having minor children and being a single adult versus two or more adults in the family are associated with greater risk for cancer‐related financial hardship.

## Supporting information


Table S1.
Table S2.

Table S3.

## Data Availability

Data were obtained from the 2015‐2018 National Health Interview Surveys (NHIS). These data are publicly available.
