# Supplementary material for: Comparisons of financial hardship in cancer care by family structure and among those with and without minor children using nationally representative data
Source: Cancer Med. 2024 Mar 23;13(6):e7088. doi: 10.1002/cam4.7088 (PMC10960158; doi:10.1002/cam4.7088)
Supplement: Supplementary file 1 — Table S1. Table S2. [file CAM4-13-e7088-s001.docx]

**Supplemental Table 1** Measures of healthcare-related financial hardship and their operationalization in this study, National Health Interview Surveys 2015-2019

| **Dimension** | **NHIS Question** | **Operationalization / Outcome Coding Logic** |
| --- | --- | --- |
| **Material Hardship** | **In the past 12 months, did you/anyone in the family have problems paying or were unable to pay any medical bills?** | If either of these questions were answered as ‘yes’ then material hardship=’yes’  If both were answered as ‘no’ then material hardship=’no’ |
|  | **Do you/Does anyone in your family currently have any medical bills that are being paid off over time?** |  |
| **Psychological Hardship** | **P1: How worried are you right now about not being able to pay medical costs of a serious illness or accident?** | If either P or P2 = ‘very worried’ or ‘moderately worried’ then psychological hardship=’yes’  If both P1 and P2 = ‘not too worried’ or ‘not worried at all’ then psychological hardship=’no’ |
|  | **P2: How worried are you right now about not being able to pay medical costs for normal healthcare?** |  |
| **Delaying / Foregoing Care** | **During the past 12 months, has medical care been delayed for [you/anyone in the family] because of worry about the cost?** (Do not include dental care)” | If either of these questions were answered as ‘yes’ then delay/forego care=’yes’  If both were answered as ‘no’ then delay/forego =’no’ |
|  | **During the past 12 months, was there any time when [you/someone in the family] needed medical care, but did not get it because [you/the family] couldn't afford it?** |  |
| **Reducing Prescription Costs** | **DURING THE PAST 12 MONTHS, was there any time when you needed any of the following, but didn't get it because you couldn't afford it?**  ... Prescription medicines  **DURING THE PAST 12 MONTHS, were any of the following true for you?**  …You skipped medication doses to save money  …you took less medicine to save money  …You delayed filling a prescription to save money  …You asked your doctor for a lower cost medication to save money.  …You bought prescription drugs from another country to save money.  …You used alternative therapies to save money. | If any of these questions were answered as ‘yes’ then skipping/stretching prescriptions=’yes’  If all of these questions were answered as ‘no’ then skipping/stretching prescriptions=’no’ |
| **Skipping Specialist / Skipping Follow-up Care** | **During the past 12 months, was there any time when you needed any of the following, but did not get it because you couldn't afford it**  ... To see a specialist  ... Follow-up care | If one of these questions was answered as ‘yes’ then skipping specialist / follow-up =’yes’  If both questions were answered as ‘no’ then skipping specialist / follow-up =’no’ |

**Supplemental Table 2** Characteristics of individuals without cancer by family status, restricted to participants aged <60 years, survey-weighted frequencies, N=73,437, National Health Interview Surveys 2015-2019

| **Characteristic** | **Everyone** | **Two or more adults in family / without minor children** | **Two or more adults in family / with minor children** | **Single adult in family / without minor children** | **Single adult in family / with minor children** |
| --- | --- | --- | --- | --- | --- |
|  | **(N=73,437)** | **(N= 21,953)** | **(N=24,618)** | **(N=21,362)** | **(N=5,504)** |
|  |  |  |  |  |  |
|  | **% (95% CI)** | **% (95% CI)** | **% (95% CI)** | **% (95% CI)** | **% (95% CI)** |
| **Age group** | | | | |  |
| <40 | 53.4 (52.8, 54.0) | 45.5 (44.6, 46.3) | 57.7 (56.9, 58.6) | 54.9 (53.7, 56.2) | 60.2 (58.7, 61.8) |
| 40-49 | 22.5 (22.1, 22.9) | 17.0 (16.4, 17.6) | 30.8 (30, 31.5) | 17.0 (16.3, 17.7) | 29.6 (28.2, 31.0) |
| 50-59 | 24.1 (23.7, 24.5) | 37.6 (36.8, 38.3) | 11.5 (11.0, 12.0) | 28.1 (27.1, 29.0) | 10.2 (9.3, 11.1) |
| **Sex** |  |  |  |  |  |
| Female | 51.7 (51.3, 52.2) | 50.7 (49.9, 51.4) | 53.6 (52.8, 54.4) | 42.9 (42.0, 43.8) | 83.3 (82.2, 84.5) |
| Male | 48.3 (47.8, 48.7) | 49.3 (48.6, 50.1) | 46.4 (45.6, 47.2) | 57.1 (56.2, 58.0) | 16.7 (15.5, 17.8) |
| **Race/Ethnicity** | | | | |  |
| Hispanic | 16.2 (15.3, 17.2) | 13.8 (12.8, 14.8) | 21.9 (20.6, 23.2) | 11.4 (10.6, 12.2) | 19.7 (18.0, 21.4) |
| Non-Hispanic Black | 13.9 (13.1, 14.6) | 10.9 (10.2, 11.7) | 10.2 (9.6, 10.9) | 17.2 (16.1, 18.3) | 29.0 (27.0, 31.0) |
| Non-Hispanic other | 7.4 (6.9, 7.8) | 6.9 (6.4, 7.5) | 8.2 (7.6, 8.8) | 7.7 (7.1, 8.4) | 3.8 (3.1, 4.5) |
| Non-Hispanic white | 62.6 (61.4, 63.7) | 68.3 (67.0, 69.6) | 59.7 (58.3, 61.1) | 63.7 (62.3, 65.0) | 47.5 (45.5, 49.6) |
| **Income to poverty line (PL) ratio** | | | | |  |
| < PL | 15.8 (15.2, 16.4) | 5.8 (5.4, 6.3) | 11.1 (10.5, 11.7) | 25.7 (24.5, 26.9) | 37.4 (35.8, 39.1) |
| 1-<2 x PL income | 17.4 (16.9, 17.8) | 12.0 (11.4, 12.6) | 19.8 (19.0, 20.5) | 17.4 (16.6, 18.1) | 28.0 (26.6, 29.4) |
| 2-<4 x PL income | 27.1 (26.7, 27.6) | 25.3 (24.6, 26.1) | 30.7 (29.9, 31.5) | 26.1 (25.3, 26.9) | 22.5 (21.2, 23.9) |
| >=4 x PL income | 39.7 (38.8, 40.6) | 56.8 (55.7, 57.9) | 38.5 (37.3, 39.7) | 30.9 (29.6, 32.1) | 12.0 (10.9, 13.1) |
| **Education** | | | | |  |
| No high-school degree | 8.0 (7.7, 8.4) | 4.7 (4.4, 5.1) | 6.8 (6.3, 7.2) | 10.2 (9.6, 10.8) | 18.6 (17.3, 19.8) |
| No college degree | 48.1 (47.2, 48.9) | 45.9 (44.9, 46.9) | 44.1 (43.0, 45.2) | 52.4 (51.1, 53.6) | 57.6 (56.0, 59.3) |
| At least college degree | 43.9 (43.0, 44.9) | 49.4 (48.3, 50.4) | 49.1 (47.9, 50.3) | 37.4 (36.1, 38.8) | 23.8 (22.4, 25.2) |
