# Supplementary material for: Comparisons of financial hardship in cancer care by family structure and among those with and without minor children using nationally representative data
Source: Cancer Med. 2024 Mar 23;13(6):e7088. doi: 10.1002/cam4.7088 (PMC10960158; doi:10.1002/cam4.7088)
Supplement: Supplementary file 2 — Table S3. [file CAM4-13-e7088-s002.docx]

**Supplemental Table 3** Intersectional analysis among those with cancer: financial hardships by age, divorced/separated marital status (yes versus no) and presence of minor children: National Health Interview Surveys 2015-2018

| **Outcome** | **Age<40 years** | | | | **Age 40-59 years** | | | | **Age>59 years †, #** | |
| --- | --- | --- | --- | --- | --- | --- | --- | --- | --- | --- |
|  | **Divorced / separated** | | **Not divorced / separated** | | **Divorced / separated** | | **Not divorced / separated** | | **Divorced / separated** | **Not divorced / separated** |
|  | **With minor children, N=50** | **With or without minor children*, N=77** | **With minor children, N=293** | **Without minor children, N=204** | **With minor children, N=146** | **Without minor children, N=495** | **With minor children, N=451** | **Without minor children, N=1,175** | **Without minor children, N=1,088** | **Without minor children, N=5,355** |
|  | **%**  **(95% CI)** | **%**  **(95% CI)** | **%**  **(95% CI)** | **%**  **(95% CI)** | **%**  **(95% CI)** | **%**  **(95% CI)** | **%**  **(95% CI)** | **%**  **(95% CI)** | **%**  **(95% CI)** | **%**  **(95% CI)** |
| **Material hardship** | 54.6  (41.9-67.3) | 38.8  (28.2-49.4) | 48.2  (42-54.4) | 40.9  (34.4-47.4) | 45.4  (39.0-51.8) | 44.1  (39.4-48.7) | 43.0  (38.3-47.7) | 36.5  (33.3-39.6) | 20.4  (17.7-23) | 13.6  (12.6-14.6) |
| **Psychological hardship** | 70.6  (57.3-83.9) | 70.5  (60.2-80.9) | 64.1  (58.2-70) | 60.9  (54.4-67.4) | 69.6  (62.1-77.1) | 70.9  (66.6-75.2) | 64.0  (59.0-68.9) | 60.8  (57.9-63.8) | 44.5  (41.2-47.8) | 33.1  (31.7-34.6) |
| **Delaying / foregoing care** | 28.2  (19.4-36.9) | 22.0  (15.2-28.8) | 20.0  (15.4-24.6) | 28.1  (22.6-33.6) | 29.8  (21.2-38.3) | 28.9  (24.8-33.0) | 22.8  (18.8-26.8) | 20.4  (17.7-23) | 13.1  (11.1-15.1) | 5.5  (4.8-6.2) |
| **Reducing prescription costs** | 52.8  (38.4-67.2) | 42.3  (31.0-53.6) | 42.1  (35.5-48.8) | 36.5  (29.5-43.4) | 48.3  (39.6-56.9) | 45.6  (40.4-50.8) | 31.2  (26.2-36.1) | 32.9  (29.8-36.1) | 28.3  (25.2-31.4) | 22.2  (20.9-23.5) |
| **Skipping specialist or follow-up care** | 29.4  (18.3-40.6) | 21.6  (13.9-29.3) | 13.7  (8.9-18.4) | 16.2  (10.5-21.8) | 12.1  (6.4-17.9) | 16.1  (12.3-19.9) | 8.5  (5.5-11.5) | 7.3  5.5-9.1) | 5.0  (3.2-6.8) | 2.0  (1.5-2.5) |

* Age group <40 years: sample size too small (N=27) when restricting to those without minor children

**†** The age group >59 years was not included in main model due to violation of positivity assumption regarding child-rearing. This positivity assumption does not apply in the intersectional comparison.

# The age group >59 years with minor children is not displayed due to small sample size and because most minor children in these households likely have a grandchild-grandparent rather than a child-guardian relationship with participants.
